# Change in Cardiorespiratory Fitness and the Risk of Colorectal and Prostate Cancer Incidence in Men

**DOI:** 10.1002/cam4.70430

**Published:** 2024-12-02

**Authors:** Emil Bojsen‐Møller, Kate A. Bolam, Daniel Väisänen, Sofia Paulsson, Magnus Lindwall, Helene Rundqvist, Jenny Nyberg, Maria Åberg, Elin Ekblom‐Bak

**Affiliations:** ^1^ Department of Physical Activity and Health The Swedish School of Sport and Health Sciences Stockholm Sweden; ^2^ Cardiometabolic Health and Exercise Physiology Laboratory Baker Heart and Diabetes Institute Melbourne Australia; ^3^ Research Department HPI Health Profile Institute Stockholm Sweden; ^4^ Department of Psychology University of Gothenburg Stockholm Sweden; ^5^ Department of Neurobiology, Care Sciences and Society Karolinska Institutet Stockholm Sweden; ^6^ Department of Laboratory Medicine Karolinska Institutet Stockholm Sweden; ^7^ Region Västra Götaland Sahlgrenska University Hospital Gothenburg Sweden; ^8^ Section for Clinical Neuroscience, Institute of Neuroscience and Physiology, Sahlgrenska Academy University of Gothenburg Gothenburg Sweden; ^9^ School of Public Health and Community Medicine, Institute of Medicine University of Gothenburg Gothenburg Sweden; ^10^ Region Västra Götaland Gothenburg Sweden

**Keywords:** cancer risk factors, colorectal cancer, prostate cancer, registries

## Abstract

**Introduction:**

The aim of this prospective cohort study was to investigate the associations between changes in cardiorespiratory fitness (CRF) and the risk of colorectal and prostate cancer in men.

**Methods:**

Data from men who completed a health assessment both in military conscription in youth and an occupational health profile assessment (HPA) later in life were used. CRF was assessed as estimated V̇O_2max_, using a cycle ergometer fitness test at both time points. We linked the assessment data to national register data on colorectal and prostate cancer incidence, and hazard ratios and confidence intervals were estimated using Cox proportional hazard regression.

**Results:**

139,764 men with a mean age of 18 (SD 0.6) at conscription and 43 (SD 8.9) at HPA were included. The average time between the two assessments was 25.9 (SD 9.0) years and mean follow‐up time following HPA test was 10.0 (SD 5.6) years for prostate and colorectal cancer. Annual percentage change in relative and absolute V̇O_2max_ from conscription to HPA was inversely associated with the risk of colorectal cancer incidence, hazard ratio of 0.83 (95% CI [0.72–0.94]) and 0.88 (95% CI [0.79–0.99]), respectively. These associations were driven by data from individuals in the lowest and moderate level fitness tertials at conscription. Change in CRF was not associated with prostate cancer incidence risk.

**Conclusions:**

Changes in, not only level of, CRF from youth to adulthood are related to colorectal cancer incidence risk and therefore, improving CRF should be considered as an important colorectal cancer risk reduction strategy.

## 1 Introduction

Colorectal and prostate cancer are among the most common cancers worldwide with 1.8 million and 1.4 million new cases estimated to have been diagnosed in 2020, respectively, with the prevalence expected to increase [1]. This escalating trend not only leads to devastating morbidity and mortality but also poses substantial economic burden and stress to the individual due to treatment and medical costs or inability to work [2] and to society due to their increased reliance on healthcare systems. To address this problem, identifying modifiable lifestyle factors associated with colorectal and prostate cancer risk must be prioritised.

Among these factors, a substantial body of evidence indicates that a high level of cardiorespiratory fitness (CRF) plays a pivotal role in reducing the risk of both all‐cause cancer mortality and cancer incidence [3, 4, 5]. We investigated this in this sample and found a beneficial association between higher CRF measured at one time‐point and colon cancer incidence but not for prostate cancer incidence [3]. CRF depends partly on an individual's genetics [6], but also their present level of physical activity [7]. Emerging research suggests that aerobic exercise may have a positive impact on various mechanisms known to be associated with elevated cancer risk, such as improving insulin sensitivity, mitigating systemic inflammation and addressing dyslipidaemia [8]. Despite these insights, a significant gap exists in our understanding of the lifelong trajectories of CRF and its relationship to risk of developing specific cancer types. Although a limited number of studies have explored the link between changes in CRF [9, 10, 11], these studies have primarily focused on general cancer incidence and mortality, and the time between CRF assessments has generally been shorter than a decade. Not only are different cancer types different in their pathophysiology but the mechanisms between physical activity and thus CRF are different between cancer types [12]. It is imperative that each cancer type is examined separately to better understand the relationship between CRF and specific risks for each cancer type. Given the high prevalence of prostate and colorectal cancer in Sweden and globally, the aim of this study was to examine the association between changes in CRF, assessed during youth and adulthood and the risk of colorectal and prostate cancer incidence. We hypothesised that there would be inverse associations between changes in CRF and risk of colorectal cancer but no associations for prostate cancer.

## 2 Methods

This is a prospective register‐based observational study of men who completed military conscription assessments between 1972 and 2005 in youth between the ages of 16 and 29, and later occupational health assessments in adulthood. Individual‐level data were subsequently linked to data from Statistics Sweden, the Swedish National Patient Register and the National Cause of Death register [13], to obtain relevant outcome information. Data were linked using the Swedish personal identification number. The dataset includes information collected until December 31 2021. The study was approved by the Swedish authority for ethical permissions, EPN Dnr 462‐14 with addendums Dnr 2020–03667, Dnr 2021–03310, Dnr 2021‐05638‐02 and Dnr 2023‐04937‐02 and adhered to the Declaration of Helsinki [14]. This study follows the STROBE reporting guidelines for observational studies.

The Swedish military conscription register contains data from the conscription assessment, which was compulsory for all Swedish men between 1968 and 2005. At conscription, data on anthropometrics, CRF, muscle strength, cognitive function and mental health were obtained using a standardised protocol and have been described previously [15]. Reasons for exclusions from the Swedish conscription and/or military services are numerous and described in this publication [15].

Occupational health profile assessments (HPA) have been performed since the 1970s, and the data have been stored in a central database since 1982. The data were collected from 1986 to 2020, with most tests performed after 2000. A HPA is offered to employees of companies or organisations connected to occupational or other health services, free of charge and optional for the employee. The HPA contains measurements on anthropometrics, CRF, lifestyle factors, perceived health, physical activity habits and an in‐depth interview with an HPA coach. HPI Health Profile Institute has been responsible for the development and standardisation of the methods, data collection, education of test leaders and administration of the central database throughout the years [3, 16, 17]. People were included in this study if they were men, had data for all covariates included in the full analyses, and at least two valid CRF tests, at least 1 year apart, one from conscription and one from the HPA. For participants who had two HPA tests, only their latest test data were included in the analyses. Participants were excluded if they had already been diagnosed with colorectal or prostate cancer or any of the more common cancer types (ICD 10 codes C18‐21, C25, C34, C43‐44, C61‐62, C64, C67, C71, C77, C85) for their respective analyses, prior to their HPA assessment.

### 2.1 Assessment of Cardiorespiratory Fitness

At conscription, CRF was assessed using a cycle ergometer test to measure maximal aerobic workload in units of Watts (W_max_). Two similar, yet different test procedures were used to assess CRF in the conscription assessments [15]. Absolute estimated maximal oxygen consumption (V̇O_2max_) in L∙min^−1^ was subsequently calculated as described by Nordesjö (1974), and relative V̇O_2max_ in mL∙kg^−1^∙min^−1^ was calculated thereafter. See Data S1 for formulas for the calculations that were used during the conscription assessments.

During the occupational HPA, CRF was determined using the Åstrand standardised submaximal cycle ergometer test [18]. Participants were requested to refrain from vigorous physical activity the day before, consuming a heavy meal 3 h before and smoking/using snuff 1 h before the test. In adult populations, a validation study showed small, nonsignificant mean differences at a group level (−0.07 L∙min^−1^; 95% CI, −0.21 to 0.06 L∙min^−1^) between V̇O_2max_ using the Åstrand test and directly measured V̇O_2max_ from maximal effort tests on a treadmill, with an absolute error and coefficient of variation similar to other submaximal tests (standard error of estimate, 0.48 L∙min^−1^; coefficient of variation, 18.1%) [19].

Annual percentage change of absolute and relative CRF from conscription assessment to HPA was calculated. In addition, to account for the differences in tests used at the two time points, standardised changes for both relative and absolute CRF were calculated. At conscription, a standardised score (*z*‐score) was calculated using the V̇O_2max_ mean and standard deviation from each year of conscription. At the HPA, a *z*‐score based on the mean and standard deviation for each 5‐year age interval at HPA was calculated. Changes in the *z*‐score from conscription determined the standardised change. The *z*‐score was used to examine CRF changes relative to the men's age‐matched peers. In addition, we divided participants into tertials according to their relative CRF at conscription: lowest tertial (< 44.7 mL∙kg^−1^∙min^−1^), moderate tertial CRF (44.7–50.5 mL∙kg^−1^∙min^−1^) and highest tertial (> 50.5 mL∙kg^−1^∙min^−1^).

### 2.2 Cancer Incidence

The Swedish National Patient Register and the National Cause of Death Register were used to gather information on cancer diagnoses [13]. All ICD 9 codes were converted to ICD 10 codes. ICD 10 codes C18, C19, C20 and C21 were used for colorectal cancer and C61 for prostate cancer. All participants were followed from the date of the last HPA to the date of their prostate or colorectal cancer diagnosis, death (of any cause) or until the 31 December 2021. Mortality analyses were not performed due to too few deaths where prostate or colorectal cancer was recorded as the primary cause of death.

### 2.3 Covariates

The highest level of education attained (length of education: < 9 years to postgraduate education) at the time of the HPA was drawn from Statistics Sweden by linking the participant's Swedish personal identification number. Smoking was self‐reported using the following statements: ‘I smoke… with the alternatives: At least 20 cig day^−1^, 11–19 cig day^−1^, 1–10 cig day^−1^, occasionally or never.’ Body mass index (BMI) was calculated as weight in kilogrammes divided by height in meters squared [20]. Cardiovascular disease (CVD) co‐morbidity was defined as life‐time presence of cardiovascular disease (ICD10; I10‐I13, I20‐I25, I60‐I69) and coded as yes or no.

### 2.4 Data Processing

All individuals with more or less than three standard deviations from the V̇O_2max_ mean at conscription were considered as outliers and excluded.

### 2.5 Statistical Analysis

Associations between CRF at the single time points of conscription and at HPA, and changes between the two timepoints and cancer incidence were investigated fitting cox proportional hazard regression models with age as the time scale. For the single time point analyses, four models were created; model 1 included year of test, model 2 added education level reached at HPA, model 3 further included BMI at the test point (conscription or HPA) and model 4 further included CVD co‐morbidity. For the change analyses, three models were created; model 1 was adjusted for conscription V̇O_2max_, model 2 additionally included education and model 3 further included BMI at HPA and CVD co‐morbidity.

For all models, the proportional hazard assumption was checked using scaled Schoenfeld residuals, and hazard ratios and 95% confidence intervals were extracted. For visualisation purposes, restricted cubic splines with knots at 5th, 50th and 95th percentiles were performed for all CRF outcomes with 50th percentile as the reference. All statistical analyses were performed using RStudio, version 4.2.1 (2022‐06‐23) [21]. Data wrangling was performed using the Tidyverse [22]. The survival package was used to create cox proportional hazard regression models [23] and ggplot2 [24] and survminer [25] were used for the restricted cubic splines.

## 3 Results

After integrating the Swedish military conscription register with the HPA database, we identified 142,878 individuals with CRF assessments across both databases. Of these, 731 individuals were excluded due to cancer diagnoses prior to the HPA. An additional 13 people were excluded for having an interval of less than 1 year between their conscription and HPA assessments. Furthermore, 1557 individuals were excluded due to the absence of data for one or more covariates. Lastly, 813 individuals were excluded based on their CRF values being either below or above three standard deviations from the mean recorded at conscription. A flow chart of study inclusion is included in Figure S1 within the supplement file. In total, 139,764 men were included in the analysis with a mean age of 18 years (SD 0.6 years) at conscription and 43 years (SD 8.9 years) at the HPA. The average absolute V̇O_2max_ was 3.29 L∙min^−1^ (SD 0.43 L∙min^−1^) at conscription and 3.10 L∙min^−1^ (SD 0.75 L∙min^−1^) at the HPA. The average relative V̇O_2max_ was 47.47 mL∙kg^−1^∙min^−1^ (SD 6.70 mL∙kg^−1^∙min^−1^) at conscription and 36.47 mL∙kg^−1^∙min^−1^ (SD 9.86 mL∙kg^−1^∙min^−1^) at the HPA (see Table 1 for sample characteristics). In the analytic sample, 442 colorectal and 1387 prostate cancer cases were identified during the follow‐up period.

**TABLE 1:** Study participant characteristics in relation to cardiorespiratory fitness level at conscript.

	All	Lowest tertial, < 44.7, (mL∙kg^−1^∙min^−1^)	Moderate tertial, 44.7 to 50.5, (mL∙kg^−1^∙min^−1^)	Highest tertial, > 50.5, (mL∙kg^−1^∙min^−1^)
	*N* = 139,764	*N* = 46,592	*N* = 46,583	*N* = 46,590
Age at conscript (years), mean (SD)	18.27 (0.64)	18.34 (0.77)	18.27 (0.60)	18.21 (0.51)
Age at HPA (years), mean (SD)	43.25 (8.93)	42.12 (9.03)	43.67 (9.06)	43.95 (8.60)
BMI at conscript (kg∙m^−2^), mean (SD)	21.76 (2.68)	23.26 (3.25)	21.37 (2.04)	20.65 (1.78)
BMI at HPA (kg∙m^−2^), mean (SD)	26.57 (3.84)	27.97 (4.41)	26.31 (3.45)	25.44 (3.11)
College/University degree, *n* (%)	58,609 (41.9)	15,853 (34.01)	19,485 (41.83)	23,271 (49.95)
Never smoked, *n* (%), (*N* = 107,338)	90,631 (84.4)	28,722 (81.0)	30,144 (84.2)	31,765 (88.0)
CVD co‐morbidity, *n* (%)	42,080 (30.1)	14,802 (31.8)	13,974 (30.0)	13,304 (28.6)
Absolute V̇O_2_max at Conscript (L∙min^−1^), mean (SD)	3.29 (0.43)	3.03 (0.38)	3.29 (0.36)	3.56 (0.38)
Absolute V̇O_2_max at HPA (L∙min^−1^), mean (SD)	3.10 (0.75)	3.06 (0.72)	3.09 (0.75)	3.16 (0.76)
Relative V̇O_2_max at Conscript (mL∙kg^−1^∙min^−1^), mean (SD)	47.47 (6.70)	40.15 (3.97)	47.64 (1.63)	54.64 (3.33)
Relative V̇O_2_max at HPA (mL∙kg^−1^∙min^−1^), mean (SD)	36.47 (9.86)	33.81 (9.23)	36.45 (9.56)	39.15 (10.03)
Time between tests (years), mean (SD)	25.91 (8.97)	24.70 (9.06)	26.33 (9.09)	26.69 (8.64)
Follow‐up colorectal cancer (years), mean (SD)	9.98 (5.62)	9.77 (5.59)	10.02 (5.62)	10.11 (5.64)
Follow‐up prostate cancer (years), mean (SD)	9.97 (5.62)	9.78 (5.59)	10.04 (5.62)	10.13 (5.64)
Colorectal cancer cases, *n* (%)	442 (0.32)	157 (0.34)	166 (0.35)	119 (0.26)
Prostate cancer cases, *n* (%)	1387 (0.99)	463 (0.99)	492 (1.05)	432 (0.94)
Colorectal cancer deaths, *n* (%)	118 (0.08)	40 (0.09)	53 (0.11)	25 (0.05)
Prostate cancer deaths, *n* (%)	34 (0.02)	10 (0.02)	13 (0.03)	11 (0.02)

*Note:* Tertials are calculated at conscription.

Abbreviations: BMI: body mass index, CVD: cardiovascular disease, HPA: health profile assessment, V̇O_2_max: estimated maximal oxygen consumption.

Within the single time point analyses, at conscription, both continuous relative (per mL∙kg^−1^∙min^−1^) and absolute (per L∙min^−1^) V̇O_2max_ were inversely associated with colorectal cancer incidence (Table 2). On the contrary, relative and absolute V̇O_2max_ were positively associated with prostate cancer incidence. At HPA, relative V̇O_2max_ was inversely associated with colorectal cancer incidence. Absolute V̇O_2max_ was only inversely associated with colorectal cancer incidence when adjusting for BMI (model 3) but not in the fully adjusted model 4. Both relative and absolute V̇O_2max_ measured during the HPA were positively associated with prostate cancer incidence (Table 2).

**TABLE 2:** Associations between cardiorespiratory fitness at conscription and at HPA and colorectal and prostate cancer incidence.

	Model 1		Model 2		Model 3		Model 4	
	HR	95% CI	HR	95% CI	HR	95% CI	HR	95% CI
CRF at conscription								
Colorectal cancer incidence								
Relative V̇O_2_max (mL∙kg^−1^∙min^−1^)	0.98 **	0.96–0.99	0.98 **	0.96–0.99	0.98 **	0.96–0.99	0.98 *	0.96–1.00
Absolute V̇O_2_max (L∙min^−1^)	0.77 *	0.61–0.97	0.77 *	0.61–0.97	0.73 *	0.57–0.93	0.76 *	0.59–0.96
Prostate cancer incidence								
Relative V̇O_2_max (mL∙kg^−1^∙min^−1^)	1.01 **	1.00–1.02	1.01 *	1.00–1.02	1.01 *	1.00–1.02	1.01 *	1.00–1.02
Absolute V̇O_2_max (L∙min^−1^)	1.14	1.00–1.30	1.14	0.99–1.31	1.17 *	1.01–1.35	1.17 *	1.01–1.35
CRF at HPA								
Colorectal cancer incidence								
Relative V̇O_2_max (mL∙kg^−1^∙min^−1^)	0.98 **	0.97–0.99	0.98 **	0.97–0.99	0.98 *	0.97–1.00	0.99	0.98–1.00
Absolute V̇O_2_max (L∙min^−1^)	0.87	0.75 –1.00	0.87	0.75–1.00	0.86 *	0.75–1.00	0.90	0.78–1.03
Prostate cancer incidence								
Relative V̇O_2_max (mL∙kg^−1^∙min^−1^)	1.01 ***	1.01–1.02	1.01 ***	1.01–1.02	1.01 ***	1.00–1.02	1.01 ***	1.00–1.02
Absolute V̇O_2_max (L∙min^−1^)	1.13 **	1.05–1.23	1.11 *	1.02–1.21	1.12 **	1.03–1.21	1.13 **	1.04–1.23

*Note:* Model 1: year of test, Model 2: + education, Model 3: + BMI at the test (conscript or HPA), and Model 4: + cardiovascular disease co‐morbidity.

* *p* < 0.05.

** *p* < 0.01.

*** *p* < 0.001.

**FIGURE 1:**
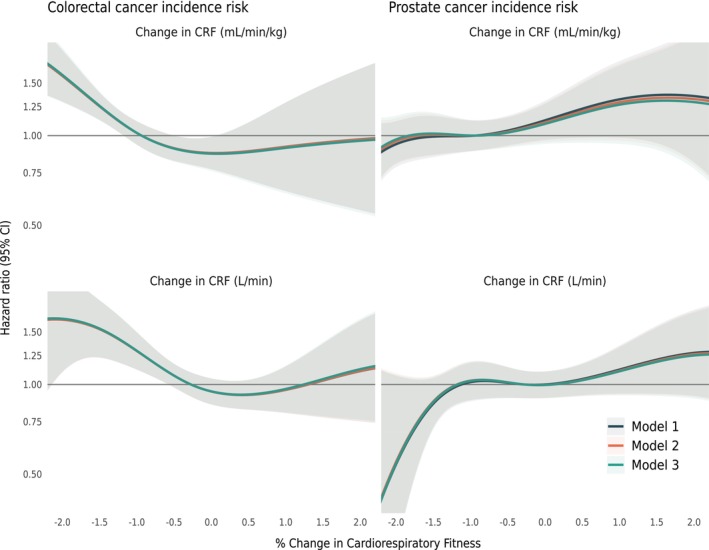
Restricted cubic splines of the cox proportional hazard model examining the association between annual percentage change in relative V̇O_2max_ (mL∙kg^−1^∙min^−1^, upper figures) and absolute V̇O_2max_ (L∙min^−1^, bottom figures). Model 1: Baseline V̇O_2max_, Model 2: + education, Model 3: + body mass index (BMI) and cardiovascular disease co‐morbidity.

**FIGURE 2:**
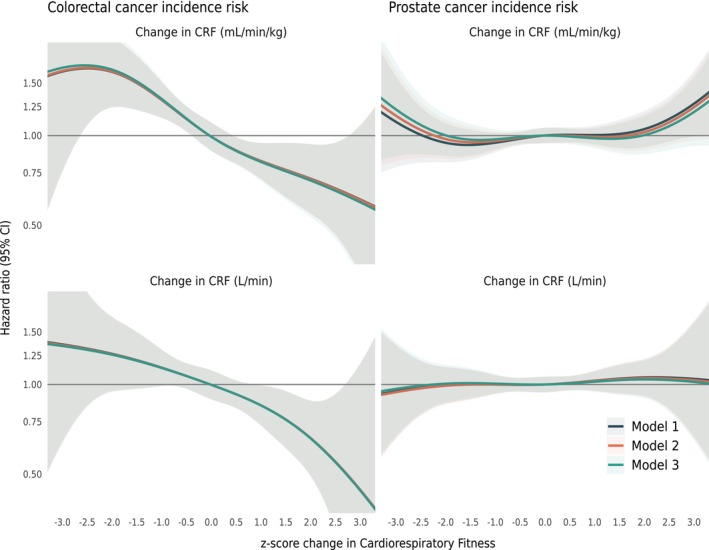
Restricted cubic splines of the cox proportional hazard model examining the association between standardised change in relative V̇O_2max_ (mL∙kg^−1^∙min^−1^, upper figures) and absolute V̇O_2max_ (L∙min^−1^, bottom figures). Model 1: Baseline V̇O_2max_, Model 2: + education, Model 3: + body mass index (BMI) and cardiovascular disease co‐morbidity. *Z*‐scores at conscription are calculated with the mean and standard deviation from each year due to change in test methods. *Z*‐score at health profile assessment (HPA) is calculated with the mean and standard deviation from conscription.

Change in V̇O_2max_ was inversely associated with colorectal cancer incidence with no major deviations between the different ways in which change in V̇O_2max_ was expressed (Table 3). Hazard ratios ranged from 0.82 (95% CI [0.73–0.92]) to 0.88 (95% CI [0.79–0.99]) for the fully adjusted model 3 (Figures 1 and 2). Change in V̇O_2max_ was, in general, not associated with prostate cancer incidence with hazard ratios ranging from 1.02 (95% CI [0.96–1.08]) to 1.08 (95% CI [0.98–1.19]) for the fully adjusted model, with the exception of model 1 for the percentage change in relative V̇O_2max_, which did show a significant positive association (HR 1.10, 95% CI [1.00–1.20]) (Table 3). However, this association was attenuated, and no longer statistically significant in models 2 to 3.

**TABLE 3:** Associations between change in cardiorespiratory fitness between conscription and HPA, and colorectal and prostate cancer incidence.

	Model 1		Model 2		Model 3	
	HR	95% CI	HR	95% CI	HR	95% CI
Colorectal cancer incidence						
Annual % change in relative V̇O_2_max (mL∙kg^−1^∙min^−1^)	0.82 **	0.72–0.92	0.82 **	0.72–0.92	0.83 **	0.72–0.94
Annual % change in absolute V̇O_2_max (L∙min^−1^)	0.88 *	0.78–0.99	0.88 *	0.78–0.99	0.88 *	0.79–0.99
Change in relative V̇O_2_max z‐score_age5years_	0.81 ***	0.73–0.90	0.81 ***	0.73–0.90	0.82 ***	0.73–0.92
Change in absolute V̇O_2_max z‐score_age5years_	0.85 **	0.77–0.95	0.85 **	0.77–0.95	0.86 **	0.78–0.96
Prostate cancer incidence						
Annual % change in relative V̇O_2_max (mL∙kg^−1^∙min^−1^)	1.10 *	1.00–1.20	1.08	0.98–1.18	1.08	0.98–1.19
Annual % change in absolute V̇O_2_max (L∙min^−1^)	1.06	1.00–1.13	1.05	0.98–1.11	1.04	0.97–1.12
Change in relative V̇O_2_max z‐score_age5years_	1.03	0.98–1.09	1.02	0.97–1.08	1.02	0.96–1.08
Change in absolute V̇O_2_max z‐score_age5years_	1.02	0.96–1.08	1.01	0.95–1.07	1.02	0.96–1.08

*Note:* Model 1: baseline V̇O_2_max, Model 2: + education, Model 3: +BMI at the HPA and cardiovascular disease co‐morbidity.

* *p* < 0.05.

** *p* < 0.01.

*** *p* < 0.001.

When the men were grouped into tertials by CRF levels at the time of conscription, the only significant associations found were inverse associations between change in V̇O_2max_ and colorectal cancer for the individuals in the lowest (model 4; HR 0.83, 95% CI [0.69–0.99]) and moderate level (HR 0.82, 95% CI [0.68–0.98]) CRF tertials (Table 4).

**TABLE 4:** Associations between change in cardiorespiratory fitness between conscription and HPA, and colorectal and prostate cancer incidence, in relation to tertiles of CRF level at conscription.

	% change in absolute V̇O_2_max, per year (L∙kg^−1^∙min^−1^)		Change in absolute V̇O_2_max, z‐score_age5years_	
	HR	95% CI	HR	95% CI
Colorectal cancer incidence				
Lowest tertial < 44.7 mL∙kg^−1^∙min^−1^	0.87	0.73–1.04	0.83 *	0.69–0.99
Moderate tertial 44.7–50.5 mL∙kg^−1^∙min^−1^	0.90	0.73–1.11	0.82 *	0.68–0.98
Highest tertial > 50.5 mL∙kg^−1^∙min^−1^	0.88	0.69–1.11	0.94	0.877–1.14
Prostate cancer incidence				
Lowest tertial < 44.7 mL∙kg^−1^∙min^−1^	1.09	0.96–1.25	1.10	0.99–1.23
Moderate tertial 44.7–50.5 mL∙kg^−1^∙min^−1^	1.08	0.94–1.24	1.02	0.93–1.13
Highest tertial > 50.5 mL∙kg^−1^∙min^−1^	0.97	0.83–1.13	0.98	0.88–1.08

*Note:* Based on model 3: baseline V̇O_2_max, education, body mass index (BMI) at the health profile assessment (HPA), and cardiovascular disease co‐morbidity.

* *p* < 0.05.

** *p* < 0.01.

*** *p* < 0.001.

## 4 Discussion

The main finding of this large prospective, population‐based cohort study including 139,764 men with assessments of CRF in both youth and adulthood was that change in V̇O_2max_ from youth to adulthood was inversely associated with risk of colorectal cancer incidence. However, this association appears to be driven by data from individuals in the lowest and moderate level fitness tertials at conscription, where maintaining or increasing CRF, compared to decreasing CRF was associated with significantly reduced risk for colorectal cancer incidence. Change in V̇O_2max_ was not associated with prostate cancer risk. This contributes important insights to our understanding of the relationship between changes in CRF and cancer incidence.

The relationship between CRF and cancer risk has gained increased attention in recent years. We have previously reported that higher CRF in an adult population was associated with a lower risk of colon cancer, but not prostate cancer incidence [3]. These findings are in line with several other studies [4, 26, 27, 28]. Recently, Onerup et al. showed that CRF was inversely associated with colon and rectal cancer in young Swedish conscripts, but positively associated with prostate cancer incidence. This positive association between CRF and prostate cancer may be explained by increased rates of attending prostate cancer screening appointments in individuals with higher fitness levels [29]. Additionally, we recently examined the association between CRF measured at a single timepoint in the current conscript cohort and incidence of site‐specific cancer in men. In this large cohort study of 1,078,000 men, higher CRF was linearly associated with a lower risk of developing head and neck, oesophagus, stomach, pancreas, liver, colon, rectum, kidney and lung cancer [30].

The mechanisms by which CRF influences cancer risk are still poorly understood [31]. However, emerging evidence suggests that improving insulin sensitivity, modifying systemic inflammation, controlling dyslipidaemia and decreasing adipose tissue are potential mechanisms whereby physical activity may contribute to reduced risk for certain cancers [8]. Cancer types vary in pathophysiology, and it is therefore unlikely that lifestyle factors impact the risk profile for each specific cancer to the same extent or in the same way. Additionally, inherited genetic factors contribute to prostate cancer risk more than they do to colorectal cancer risk [32], and it may be that changes in CRF do not influence prostate cancer risk to the extent they may do for colorectal cancer risk. More recent research has demonstrated shared genetics between CRF and disease risk (including cancer), which may explain part of the associations found in the present study [33]. The result of the current study highlights the importance of analysing different cancer types separately.

In this study, we further investigated the moderating effect of CRF at conscription. The results revealed that the inverse association between change in CRF and colorectal cancer incidence was mainly driven by data from individuals in the lowest fitness tertial. This implies that the greatest potential for risk reduction appears among individuals with lower fitness in their youth. The results from this study also showed that an annual increase in relative CRF by 1% was associated with an 18% reduction in risk of colorectal cancer. It is an actionable and important health message that even despite being in the lowest fitness tertial at a young age, increasing your fitness can reduce the risk for colorectal cancer incidence later in life.

The clinical implications of these findings are that we can begin to extend on the cancer risk reduction recommendations, which have until now focused on physical activity. The findings from this study provide the public with more nuanced information on the important role of improving one's fitness level, particularly those with lower and moderate fitness levels in their youth, to reduce their risk of colorectal cancer.

The strengths of this study are the large sample size, the objective measurement of CRF and the linking of the different national registers. There are also some limitations that should be detailed. The fact that two different fitness tests were used at the different time points should be considered. Despite this, the tests have both shown strong associations with gold standard maximal effort tests [19], the possibility to detect change using two different tests have not been examined. although the follow‐up time of the current study was only 10 years on average, the study findings still provide novel findings on the relationship between change in CRF and cancer incidence and mortality. There is also a risk for selection bias in the current study. The entire sample consisted of employed individuals, and the CRF levels measured in the men at conscription could be considered relatively high, which may also suggest a bias selection of higher fitness individuals. Therefore, the results may not be generalisable to an unemployed or less fit population.

## 5 Conclusion

In this large study of Swedish men, change in CRF was inversely associated with the risk of colorectal, but not prostate cancer incidence. Improving CRF should be considered as an important colorectal cancer risk reduction strategy. Future trials in larger samples and with longer follow‐up periods should be prioritised to examine the associations with prostate and colorectal cancer mortality.

## Author Contributions

**Emil Bojsen‐Møller:** conceptualization (equal), data curation (lead), formal analysis (lead), methodology (equal), writing – original draft (equal), writing – review and editing (equal). **Kate A. Bolam:** conceptualization (supporting), funding acquisition (supporting), writing – original draft (equal), writing – review and editing (equal). **Daniel Väisänen:** data curation (supporting), formal analysis (supporting), writing – review and editing (supporting). **Sofia Paulsson:** data curation (supporting), methodology (supporting), resources (supporting), writing – review and editing (supporting). **Magnus Lindwall:** conceptualization (supporting), methodology (supporting), supervision (supporting), writing – review and editing (supporting). **Helene Rundqvist:** conceptualization (supporting), funding acquisition (supporting), methodology (supporting), writing – review and editing (supporting). **Jenny Nyberg:** data curation (supporting), formal analysis (supporting), resources (supporting), writing – review and editing (supporting). **Maria Åberg:** conceptualization (supporting), data curation (equal), formal analysis (supporting), writing – review and editing (equal). **Elin Ekblom‐Bak:** conceptualization (equal), formal analysis (supporting), funding acquisition (lead), methodology (equal), project administration (lead), supervision (lead), writing – original draft (supporting), writing – review and editing (supporting).

## Ethics Statement

The study was approved by the Swedish authority for ethical permissions, EPN Dnr 462‐14 with addendums Dnr 2020‐03667, Dnr 2021‐03310, Dnr 2021‐05638‐02 and Dnr 2023‐04937‐02. No consent was obtained from participants at conscription since data were retrieved from registers. The participants gave written informed consent at the HPA to participate and that their information was saved in the database. The study was performed in accordance with the Declaration of Helsinki.

## Consent

The authors have nothing to report.

## Conflicts of Interest

Author S.P. (responsible for data collection validity) is employed by HPI Health Profile Institute. The remaining authors declare no conflicts of interests.

## Supporting information


Data S1.

## Data Availability

The data underlying the findings in our study are not publicly available because the original approval from the Regional ethics board and the informed consent form did not include such direct, free access to the data. Data are owned by, and can be requested from, the HPI Health Profile Institute at support@hpihealth.se.

## References

[1] H. Sung , J. Ferlay , R. L. Siegel , et al., “Global Cancer Statistics 2020: GLOBOCAN Estimates of Incidence and Mortality Worldwide for 36 Cancers in 185 Countries,” CA: A Cancer Journal for Clinicians 71, no. 3 (2021): 209–249. 33538338 10.3322/caac.21660

[2] P. M. Carrera , H. M. Kantarjian , and V. S. Blinder , “The Financial Burden and Distress of Patients With Cancer: Understanding and Stepping‐Up Action on the Financial Toxicity of Cancer Treatment,” CA: A Cancer Journal for Clinicians 68, no. 2 (2018): 153–165. 29338071 10.3322/caac.21443 PMC6652174

[3] E. Ekblom‐Bak , E. Bojsen‐Møller , P. Wallin , et al., “Association Between Cardiorespiratory Fitness and Cancer Incidence and Cancer‐Specific Mortality of Colon, Lung, and Prostate Cancer Among Swedish Men,” JAMA Network Open 6, no. 6 (2023): e2321102. 37382952 10.1001/jamanetworkopen.2023.21102 PMC10311389

[4] A. Onerup , K. Mehlig , E. Ekblom‐Bak , L. Lissner , M. Börjesson , and M. Åberg , “Cardiorespiratory Fitness and bmi Measured in Youth and 5‐Year Mortality After Site‐Specific Cancer Diagnoses in Men—A Population‐Based Cohort Study With Register Linkage,” Cancer Medicine 12, no. 19 (2023): 20000–20014. 37732468 10.1002/cam4.6553 PMC10587926

[5] D. P. Pozuelo‐Carrascosa , C. Alvarez‐Bueno , I. Cavero‐Redondo , S. Morais , I. M. Lee , and V. Martínez‐Vizcaíno , “Cardiorespiratory Fitness and Site‐Specific Risk of Cancer in Men: A Systematic Review and Meta‐Analysis,” European Journal of Cancer 113 (2019): 58–68. 30981949 10.1016/j.ejca.2019.03.008

[6] C. Bouchard , R. Lesage , G. Lortie , et al., “Aerobic Performance in Brothers, Dizygotic and Monozygotic Twins,” Medicine and Science in Sports and Exercise 18, no. 6 (1986): 639–646. 3784876

[7] J. Zeiher , K. J. Ombrellaro , N. Perumal , T. Keil , G. B. M. Mensink , and J. D. Finger , “Correlates and Determinants of Cardiorespiratory Fitness in Adults: A Systematic Review,” Sports Medicine ‐ Open 5, no. 1 (2019): 39. 31482208 10.1186/s40798-019-0211-2 PMC6722171

[8] K. I. Avgerinos , N. Spyrou , C. S. Mantzoros , and M. Dalamaga , “Obesity and Cancer Risk: Emerging Biological Mechanisms and Perspectives,” Metabolism 92 (2019): 121–135. 30445141 10.1016/j.metabol.2018.11.001

[9] M. T. Imboden , M. P. Harber , M. H. Whaley , et al., “The Association Between the Change in Directly Measured Cardiorespiratory Fitness Across Time and Mortality Risk,” Progress in Cardiovascular Diseases 62, no. 2 (2019): 157–162. 30543812 10.1016/j.pcad.2018.12.003

[10] T. E. Robsahm , T. Heir , L. Sandvik , et al., “Changes in Midlife Fitness, Body Mass Index, and Smoking Influence Cancer Incidence and Mortality: A Prospective Cohort Study in Men,” Cancer Medicine 8, no. 10 (2019): 4875–4882. 31270954 10.1002/cam4.2383 PMC6712445

[11] P. Zhang , X. Sui , G. A. Hand , J. R. Hébert , and S. N. Blair , “Association of Changes in Fitness and Body Composition With Cancer Mortality in Men,” Medicine and Science in Sports and Exercise 46, no. 7 (2014): 1366–1374. 24276414 10.1249/MSS.0000000000000225 PMC4031307

[12] P. Hofmann , “Cancer and Exercise: Warburg Hypothesis, Tumour Metabolism and High‐Intensity Anaerobic Exercise,” Sports 6, no. 1 (2018): 10. 29910314 10.3390/sports6010010 PMC5969185

[13] H. L. Brooke , M. Talbäck , J. Hörnblad , et al., “The Swedish Cause of Death Register,” European Journal of Epidemiology 32, no. 9 (2017): 765–773. 28983736 10.1007/s10654-017-0316-1 PMC5662659

[14] World Medical Association , “World Medical Association Declaration of Helsinki: Ethical Principles for Medical Research Involving Human Subjects,” Journal of the American Medical Association 310, no. 20 (2013): 2191–2194. 24141714 10.1001/jama.2013.281053

[15] J. F. Ludvigsson , D. Berglind , K. Sundquist , J. Sundström , P. Tynelius , and M. Neovius , “The Swedish Military Conscription Register: Opportunities for Its Use in Medical Research,” European Journal of Epidemiology 37, no. 7 (2022): 767–777. 35810240 10.1007/s10654-022-00887-0 PMC9329412

[16] E. Ekblom‐Bak , Ö. Ekblom , G. Andersson , et al., “Decline in Cardiorespiratory Fitness in the Swedish Working Force Between 1995 and 2017,” Scandinavian Journal of Medicine & Science in Sports 29, no. 2 (2019): 232–239. 30351472 10.1111/sms.13328 PMC7379642

[17] E. Ekblom‐Bak , B. Ekblom , J. Söderling , et al., “Sex‐ and Age‐Specific Associations Between Cardiorespiratory Fitness, CVD Morbidity and All‐Cause Mortality in 266.109 Adults,” Preventive Medicine 1, no. 127 (2019): 105799. 10.1016/j.ypmed.2019.105799 31454664

[18] I. Astrand , “Aerobic Work Capacity in Men and Women With Special Reference to Age,” Acta Physiologica Scandinavica. Supplementum 49, no. 169 (1960): 1–92. 13794892

[19] F. Bjorkman , E. Ekblom‐Bak , O. Ekblom , and B. Ekblom , “Validity of the Revised Ekblom Bak Cycle Ergometer Test in Adults,” European Journal of Applied Physiology 116, no. 9 (2016): 1627–1638. 27311582 10.1007/s00421-016-3412-0 PMC4983286

[20] S. Pati , W. Irfan , A. Jameel , S. Ahmed , and R. K. Shahid , “Obesity and Cancer: A Current Overview of Epidemiology, Pathogenesis, Outcomes, and Management,” Cancers 15, no. 2 (2023): 485. 36672434 10.3390/cancers15020485 PMC9857053

[21] R Core Team , A Language and Environment for Statistical Computing (Vienna, Austria: R Foundation for Statistical Computing, 2013).

[22] H. Wickham , M. Averick , J. Bryan , et al., “Welcome to the Tidyverse,” Journal of Open Source Software 4, no. 43 (2019): 1686.

[23] T. M. Therneau , “TL Original S > R Port and R Maintainer, Elizabeth A, Cynthia C. survival: Survival Analysis,” 2023, https://CRAN.R‐project.org/package=survival.

[24] H. Wickham , W. Chang , L. Henry , T. L. Pedersen , K. Takahashi , and C. Wilke , “ggplot2: Create Elegant Data Visualisations Using the Grammar of Graphics,” 2023, https://CRAN.R‐project.org/package=ggplot2.

[25] A. Kassambara , M. Kosinski , and P. Biecek , “Fabian S. survminer: Drawing Survival Curves using ‘ggplot2’,” 2021, https://CRAN.R‐project.org/package=survminer.

[26] C. H. Marshall , M. H. Al‐Mallah , Z. Dardari , et al., “Cardiorespiratory Fitness and Incident Lung and Colorectal Cancer in Men and Women: Results From the Henry Ford Exercise Testing (FIT) Cohort,” Cancer 125 (2019): 2594–2601. 31056756 10.1002/cncr.32085

[27] S. G. Lakoski , B. L. Willis , C. E. Barlow , et al., “Midlife Cardiorespiratory Fitness, Incident Cancer, and Survival After Cancer in Men: The Cooper Center Longitudinal Study,” JAMA Oncology 1, no. 2 (2015): 231–237. 26181028 10.1001/jamaoncol.2015.0226 PMC5635343

[28] J. B. Peel , X. Sui , C. E. Matthews , et al., “Cardiorespiratory Fitness and Digestive Cancer Mortality: Findings From the Aerobics Center Longitudinal Study,” Cancer Epidemiology, Biomarkers & Prevention 18, no. 4 (2009): 1111–1117. 10.1158/1055-9965.EPI-08-0846 PMC2688961 19293313

[29] C. Crump , P. Stattin , J. D. Brooks , et al., “Early‐Life Cardiorespiratory Fitness and Long‐Term Risk of Prostate Cancer,” Cancer Epidemiology, Biomarkers & Prevention 29, no. 11 (2020): 2187–2194. 10.1158/1055-9965.EPI-20-0535 PMC7642066 32856610

[30] A. Onerup , K. Mehlig , A. Geijerstam , et al., “Associations Between Cardiorespiratory Fitness in Youth and the Incidence of Site‐Specific Cancer in Men: A Cohort Study With Register Linkage,” British Journal of Sports Medicine 57, no. 19 (2023): 1248–1256. 37582636 10.1136/bjsports-2022-106617 PMC10579181

[31] C. M. Friedenreich , E. Shaw , H. K. Neilson , and D. R. Brenner , “Epidemiology and Biology of Physical Activity and Cancer Recurrence,” Journal of Molecular Medicine 95, no. 10 (2017): 1029–1041. 28620703 10.1007/s00109-017-1558-9 PMC5613065

[32] P. Lichtenstein , N. V. Holm , P. K. Verkasalo , et al., “Environmental and Heritable Factors in the Causation of Cancer—Analyses of Cohorts of Twins From Sweden, Denmark, and Finland,” New England Journal of Medicine 343, no. 2 (2000): 78–85. 10891514 10.1056/NEJM200007133430201

[33] A. N. Nordeidet , M. Klevjer , U. Wisløff , M. Langaas , and A. Bye , “Exploring Shared Genetics Between Maximal Oxygen Uptake and Disease: The HUNT Study,” Physiological Genomics 55, no. 10 (2023): 440–451, 10.1152/physiolgenomics.00026.2023. 37575066

