# Supplementary material for: Change in Cardiorespiratory Fitness and the Risk of Colorectal and Prostate Cancer Incidence in Men
Source: Cancer Med. 2024 Dec 2;13(23):e70430. doi: 10.1002/cam4.70430 (PMC11609596; doi:10.1002/cam4.70430)
Supplement: Supplementary file 1 — Data S1. [file CAM4-13-e70430-s001.docx]

**Supplementary file**

Figure s1 - Flow chart of study inclusion


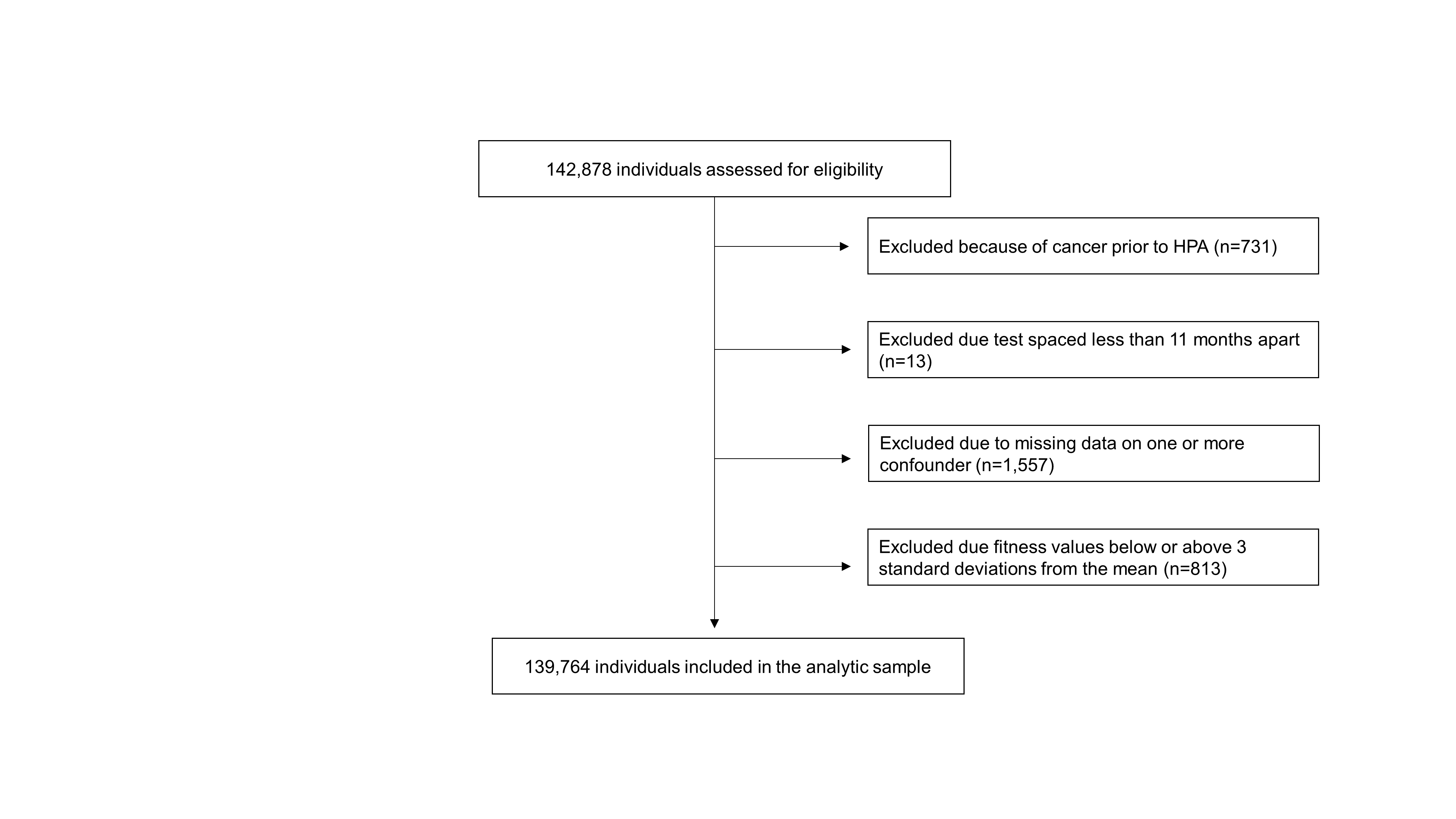


HPA: Health Profile Assessment

**Formulas**

VO_2max_ at conscription (Nordesjö (1974)):

1960 to 1983: ${VO}_{2max}= 0.01031 \times wmax \left( Watt \right)+72$

1983 to 2005: ${VO}_{2max}=\left( 0.938 \times wmax \left( Watt \right)-38.2 \right)*0.01031+0.72$

**R-script for calculating z-scores:**

**Z-score at conscription:**

data <- data %>%

group_by(year) %>% # group by year of test

mutate(z_vo2max_conscription= (vo2max_conscription-mean(vo2max_conscription)) /sd(vo2max_conscription))

# at z-score is calculated using the mean and standard deviation from conscription

**Z-score at conscription:**

data <- data %>%

group_by(age5) %>% # group by five year age intervals at HPA

mutate(z_vo2max_HPA_age5years = (vo2max_HPA-mean(vo2max_HPA))/sd(vo2max_HPA))
